# Crosstalk of Immune Cells and Platelets in an Ovarian Cancer Microenvironment and Their Prognostic Significance

**DOI:** 10.3390/ijms24119279

**Published:** 2023-05-25

**Authors:** Katarzyna Aneta Pankowska, Grażyna Ewa Będkowska, Joanna Chociej-Stypułkowska, Małgorzata Rusak, Milena Dąbrowska, Joanna Osada

**Affiliations:** Department of Haematological Diagnostics, Medical University of Bialystok, Waszyngtona 15A Street, 15-269 Bialystok, Poland

**Keywords:** ovarian cancer, immune cells, lymphocytes, NK cells, MDSC, macrophages, dendritic cells, neutrophils, platelets, prognostic

## Abstract

Ovarian cancer (OC) is one of the deadliest gynecological cancers, largely due to the fast development of metastasis and drug resistance. The immune system is a critical component of the OC tumor microenvironment (TME) and immune cells such as T cells, NK cells, and dendritic cells (DC) play a key role in anti-tumor immunity. However, OC tumor cells are well known for evading immune surveillance by modulating the immune response through various mechanisms. Recruiting immune-suppressive cells such as regulatory T cells (Treg cells), macrophages, or myeloid-derived suppressor cells (MDSC) inhibit the anti-tumor immune response and promote the development and progression of OC. Platelets are also involved in immune evasion by interaction with tumor cells or through the secretion of a variety of growth factors and cytokines to promote tumor growth and angiogenesis. In this review, we discuss the role and contribution of immune cells and platelets in TME. Furthermore, we discuss their potential prognostic significance to help in the early detection of OC and to predict disease outcome.

## 1. Introduction

Ovarian cancer (OC) is one of the biggest problems in gynecological oncology, causing over 207,000 deaths worldwide in 2020. Ovarian malignant neoplasm ranks eighth in the world among cancers in women [1,2]. It is distinguished by a poor prognosis, remarkably high death rate, and a five-year survival rate that is continuously under 45%. If a cancer is detected at an early stage (International Federation of Obstetrics and Gynecology (FIGO I)) before it spreads outside of the ovaries, the majority of women can be effectively treated using standard therapeutic approaches, with a survival rate of up to 90%. When OC spreads to the pelvic organs (FIGO II), the abdominal organs (FIGO III), and beyond the peritoneal cavity (FIGO IV), the situation dramatically changes. The poor survival rate for OC is associated with detection at an advanced stage due to the absence of distinct signs and symptoms in the initial stages of the disease and a lack of proper screening. In the majority of patients, nonspecific gastrointestinal symptoms such as flatulence, a feeling of fullness, and reproductive organ problems such as excessive vaginal bleeding or feeling pressure in the pelvis may occur more than a year before diagnosis [3,4]. This leads to approximately 70% of women being diagnosed in the advanced stages of the disease (FIGO III and IV) [5].

More than 90% of ovarian malignant neoplasms in developed nations are of an epithelial origin, with the remaining 10% coming from stromal cells (5–6%) or germ cells (2–3%) [6]. High- and low-grade serous ovarian cancers account for 70% of all epithelial-derived ovarian tumors; the remaining 30% are endometrial, mucinous, and clear cell carcinomas. Here, two lines of ovarian carcinogenesis development with the degree of histological and molecular differentiation were identified and taken into consideration. Serous (low-grade serous ovarian carcinoma (LGSOC)), clear cell, and endometrial cancers are examples of well-differentiated tumors that fall under type I and are characterized by slow development and minimal spreading potential. Serous (high-grade serous ovarian carcinomas (HGSOC)), endometrial, and sarcomas are examples of type II low-differentiated carcinomas that exhibit rapid growth and a strong metastatic potential. Over 95% of cases of HGSOC are correlated with genetic mutations in the tumor suppressor protein p53, which account for the late stage (FIGO III/IV) diagnosis of the disease when the cancer has advanced to the peritoneum [7]. Additionally, almost 85% of hereditary OC cases and more than one-fifth of all occurrences of OC are linked to mutation in genes such as BRCA1 and BRCA2 [8,9].

Combination therapy, which consists of surgery and platinum-based chemotherapy, is the recommended approach for treating OC. However, the effectiveness of this treatment varies depending on the histological nature of the tumor. Recent clinical trials suggest that drugs targeting signaling pathways, such as the PI3K- and RAS-signaling pathways, may be a new and promising option for treating OC [10]. Despite this, recurrence is seen in the majority of women within 18 months [10,11].

To gain a better understanding of OC development and progression, it is vital to recognize the functioning of the ovarian tumor microenvironment, particularly the activity and characteristics of immune cells. Various soluble factors (cytokines, chemokines, and proteins) and direct cell-to-cell interactions comprise the active network required for the development of local immunosuppression, allowing cancer cells to survive, grow, and develop metastatic qualities.

Platelets, which are derived from megakaryocytes, are anucleate cell fragments that play a significant role in primary hemostasis via their multilayer interaction between platelet and cancer cells in the tumor microenvironment, bloodstream, and peritoneal fluid. Increasing evidence in recent years suggests their crucial involvement in cancer metastatic spread, notably in the promotion of the invasiveness, angiogenesis, and anti-apoptotic activity in OC. Moreover, increasing evidence suggests that platelets may play important roles in cancer [12]. In OC, thrombocytosis, both at the time of initial diagnosis and at recurrence, has been associated with worse disease outcomes [13].

The purpose of this review is to summarize and better understand the involvement of immune cells and platelets in the development and progression of OC, as well show as their prognostic significance.

## 2. Tumor Development

The presence of leukocytes within tumors, discovered by Rudolf Virchow in the 19th century, offered the first indication of a possible relationship between inflammation and cancer. A role for inflammation in carcinogenesis is now well accepted, and an inflammatory microenvironment is an essential component of many cancers [14].

Several studies have linked ovarian surface inflammation-related factors to the development of OC [15,16]. There are two strong carcinogenesis models: ovulation theory and chronic inflammation, both of which propose that incessant ovulation or chronic exposure to external or endogenous triggers a cascade of immune cells to injury, causing damage to the ovarian endothelium, DNA damage through reactive oxygen species (ROS) release, and cytokine production, all of which increase the risk of malignant transformation. Chronic inflammation can promote and support a variety of cellular events that lead to the production or selection of aggressive cancer cells [17].

**Figure 1:**
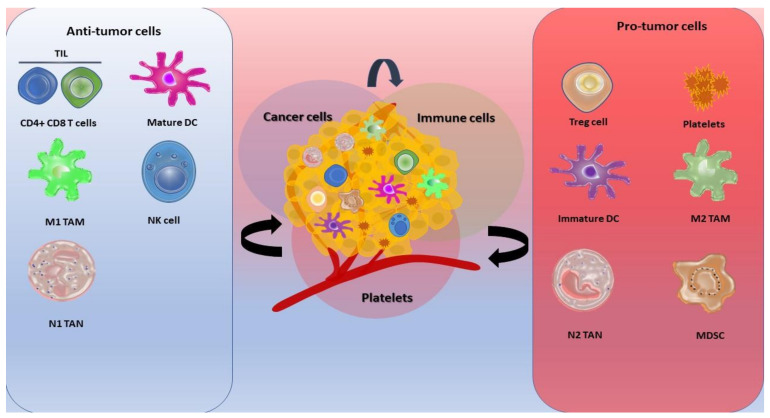
In the tumor microenvironment (TME), cells have specific components and roles. These cells actively interact with one another through the molecules they secrete (cytokines and chemokines) and receptors they express forming an evolving microenvironment. Cell components in TME can be divided into cancer cells, immune cells, and platelets. Different cell components can localize at distinct locations along the spectrum from anti-tumor (tumor infiltrating lymphocytes (TIL), mature dendritic cells (mature DC), M1 tumor-associated macrophages (M1 TAMs), natural killers cells (NK cells), and N1 tumor-associated-neutrophils (N1 TANs)) to pro-tumor effect (T regulatory cells (Treg cells), platelets, immature dendritic cells (immature DC), M2 tumor-associated macrophages (M2 TAMs), N2 tumor-associated-neutrophils (N2 TANs), and myeloid-derived suppressor cells (MDSC)), and the same group of cells may also be re-polarized depending on the signals in the TME. A single tumor site’s ability to advance or retreat is influenced by TME’s intricate cellular and the molecular regulatory network as a whole.

As previously stated, genomic instability caused by DNA damage could be one of the mechanisms of cancer initiation. A pro-inflammatory microenvironment is constantly replenished by a wide range of cytokines, growth factors, and ROS, all of which can damage DNA, switch to an antiapoptotic pathway, and trigger cancer transformation. During transformation, cells activate pro-survival signaling pathways rather than pro-apoptotic pathways that oncogenes normally activate to initiate the transformation of normal cells. The presence of an abundance of inflammatory mediators promotes a pro-inflammatory microenvironment, which can trigger the reprogramming of the surrounding cells to establish a tumor microenvironment (TME) (Figure 1) [18]. Besides malignant-transformed cells, TME is composed of normal cells including epithelial cells, fibroblasts, muscle cells, and inflammatory immune cells. Several innate and adaptive immune cells, such as macrophages, neutrophils, myeloid-derived suppressor cells (MDSCs), and T regulatory cells (Treg cells), shape the peritoneal TME directly or indirectly (via soluble interactions), creating a permissive environment for tumor development. The formation of a complex immune suppression network that effectively neutralizes anticancer activity is one of the primary causes of disease progression and therapy failure. In this part of the review, we closely focus on selected players of pro- and anti-cancer immunity in TME.

### 2.1. Tumor-Associated Macrophages

Macrophages are an essential population of immune cells that participate in inflammation and tumorigenesis. Among them, macrophages that either reside or are recruited to the tumor tissue are termed tumor-associated macrophages (TAMs) [19].

TAMs can be derived from resident macrophages or infiltrating macrophages from bone marrow monocytes circulating in the blood. TAMs can exhibit either the anti-tumor M1 macrophage or the pro-tumor M2 macrophage phenotype depending on the stimuli in the TME [20,21]. TAMs are the most prevalent type of immune cells in OC TME by volume and number alone, and numerous studies have connected tumor growth and survival to the characteristic properties of those cells [22].

Monocytes are differentiated into M1 macrophages in response to stimulation with interferon-gamma (IFN-γ), lipopolysaccharide (LPS), and granulocyte-macrophage-colony-stimulating factor (GM-CSF), which can secrete IL-1, IL-12, tumor necrosis factor (TNFα), and stromal cell-derived factor 1 (SDF-1) [23,24]. M1 macrophages possess cytotoxicity, tumor suppression, and immune-stimulation functions.

Monocytes developed into M2 macrophages when activated by cytokines such as IL-4 and IL-13. In OC, TAMs are predominantly M2 macrophages that produce and secrete anti-inflammatory/immunosuppressive cytokines such as IL-10 and transforming growth factor beta 1(TGFB1) and chemokines (CCL17, CCL18, and CCL22) associated with tumor invasion, angiogenesis, metastasis, and early recurrence [25]. Moreover, monocytes develop into M2 macrophages more quickly in an immunosuppressive environment [26,27].

TAM heterogeneity cannot be explained by a simple dichotomy of M1/M2 macrophages. Transcriptome analysis revealed a TAM spectrum model. M1 and M2 macrophages can be thought of as two endpoints of a spectrum with a wide range of functional states; TAM subpopulations in between the two ends can share characteristics of both M1 and M2 types [28,29]. Singhal et al. discovered that TAMs can co-express M1/M2 markers, together with T cell co-inhibitory and co-stimulatory receptors [30]. The dynamic nature of the TME cellular environment provides a foundation for TAM plasticity. Macrophages undergo reversible changes in their functional phenotypes and distribution in response to numerous microenvironmental cues, including tissue and tumor-specific cytokines and locally generated molecules. As a result, TAMs with varying degrees of infiltration and functional states can be found in distinct tumor histotypes and microregions of the same tumor. According to the genome-wide expression analysis of TAMs in HGSOC patients, not only are traditional M2 markers such as IL-10 and CD163 upregulated, but so are several M1 markers such as CD86 and TNF-α [31].

TAM polarization is strongly influenced by soluble and insoluble components found in the OC TME, which includes both the peritoneal and primary sites. TME is thought to stimulate TAMs to exhibit an immunosuppressive and pro-tumoral phenotype, encouraging cancer growth and progression. However, the key players in TAMs education, are the cancer cells themselves [32,33].

The TAMs pro-tumoral phenotype is also initiated by colony-stimulated factor 1 (CSF-1). CSF-1 is a key cytokine that regulates macrophage differentiation, development, and function, in addition to transforming macrophages into an M2-polarized phenotype [34]. Furthermore, cancer cells are the primary generator of CSF-1 in TME [31,32,35,36]. Colony stimulating factor 1 receptor (CSF-1R) inhibition significantly increases the proportion of M1 macrophages expressing CCR2, IL-12, and IFN-γ, decreasing the immunosuppressive state of TME [37]. Additionally, allowing monocytes to consume CSF-1 in the presence of high levels of leukemia inhibitory factor (LIF) and IL-6 in OC ascites can boost the generation of TAMs [38]. It was discovered that OC cell mucins trigger a TAMs immunosuppressive profile, as seen by the strong IL-10 production [39]. When co-cultured with OC cells, macrophages unregulated the expression of M2-associated macrophage mannose receptor (MR; CD206) [33]. MR was reported to be expressed in TAMs isolated from OC patients. TAMs are directed toward an immune-suppressive phenotype when activated by tumor mucins such as cancer antigen 125 (CA125) and tumor-associated glycoprotein 72 (TAG-72) [39]. Mucin2 (MUC2) is overexpressed in OC cells and is an independent poor prognostic factor in OC patients. MUC2 expression on tumor cells is inversely associated with TAM M1/M2 ratio and promotes cancer growth [40].

M2-like TAMs participate in OC development through their significant immunosuppressive impact on immune cells in TME. Firstly, M2 macrophages secrete transforming growth factor (TGF-α), IL-6, IL-10, CCL18, and CCL22, which attract Treg cells and promote T cell differentiation towards the Th2 phenotype [41]. Secondly, IL-10 and TGF-β inhibit the cytotoxic activity of NK cells and cytotoxic T lymphocytes (CTL). In addition, IL-10 blocks the maturation of DC. CCL18 promotes T cell anergy and unresponsiveness [41].

Another mechanism through which M2-like TAMs regulate T cell activity is their influence on the checkpoint programmed cell death protein 1 (PD-1). The interaction of PD-1 with its ligand PD-L1 on macrophages inhibits T cell activation by depleting l-arginine, which is essential for proper T cell function. Arginase I (Arg1) is a l-arginine processing enzyme that is found in M2 macrophages. Arg1 decomposes l-arginine in the TME to form l-ornithine and urea. L-arginine deficiency inhibits the re-expression of the CD3ζ chain, which is internalized by antigen stimulation and receptor (TCR) signaling. T cells become unresponsive, manifested in the inhibition of their proliferation, cytotoxicity, the production of cytokines, finally resulting in the blocking of tumor-specific T cell responses. The PI3K/AKT and Ras-MEK-ERK pathways are the main signaling pathways inhibited via triggered PD-1 [42].

Additionally, more than 70% of TAMs of OC patients are characterized by the expression of B7-H4, a coinhibitory molecule induced by IL-6 and IL-10. B7-H4 is a transmembrane protein belonging to the B7 family of costimulatory proteins. B7-H4 binds the putative receptor on activated CD4+ and CD8+ T cells, causing a decrease in their proliferation and reduction in IL-2 production [43]. Selectively blocking B7-H4 expressed on the surface of ovarian TAMs significantly increased T-cell proliferation [43]. Moreover, the intensity of B7-H4 on TAMs was associated with the number of Treg cells [40].

Exosomes produced by M2-like TAMs in the ovarian TME inhibit immunological effector activities. TAM-derived exosomes, in particular, contain a high concentration of proteins as well as DNA, mRNA, and miRNA molecules, which together suppress T cell activity and promote an imbalance between Treg cells and T helper 17 cells (Th17) cells by directly targeting signal transducer and activator of transcription 3 (STAT3) in CD4+ T cells [44].

### 2.2. Dendric Cell

Dendritic cells (DCs) operate as a bridge between the innate and adaptive immune systems by capturing endogenous or foreign antigens, processing them, and presenting the antigenic peptides to other immune cells [9,45,46,47]. Based on functional and phenotypic features, DC can be divided into two main subsets: the conventional DC (cDC) that is specialized in antigen presentation, and the plasmacytoid DC (pDC) that produces IFNγ upon antigen stimulation, aside from activating lymphocytes and other myeloid cells plasma cells [48,49].

When exposed to an antigen, DC goes through a maturation process that is marked by an increase in costimulatory molecules, downregulation of pre-existing chemokine receptors, and the acquisition of CCR7. The latter drives DC to lymph nodes through the secreted chemokines CCL19 (MIP-3β) and CCL21. Mature DC can bind with the CD40 ligand on other cells to secrete IL-12, in addition to activating naïve CD8+ T cells [45,50,51,52,53].

In addition to T cell activation, DCs are essential to the expansion of the population of CTL within TME. Intratumoral cDCs are the only type of phagocytosing tumor myeloid cell that can promote CD8+ T cell proliferation and are responsible for intratumoral CTL proliferation both in vivo and in vitro [54,55].

Unfortunately, cDC are rarely present in ovarian TME and show signs of immaturity, especially in the early stages of the disease, suggesting that they might contribute to the development of tumors [56]. Immune-suppressive milieu of OC are abundant with cytokines and inhibitory molecules, which promote the differentiation of immature DCs cells, enhance tolerance, and accelerate tumor growth [35,57,58].

Immune checkpoint signaling could play a role in DC dysfunction. OC cells may enhance PD-L1 expression in DCs by secreting (TGF-β) and prostaglandin E2 (PGE2) into TME [59]. To impair the antitumor effect, specific DCs interact with immunosuppressive cells. Inducible co-stimulator (ICOS) is expressed on immunosuppressive Treg cells and pDCs in the OC TME activate Treg cells by expressing the ICOS ligand, resulting in tumor progression [60].

In OC, insulin-like growth factor (IGF) also affects DCs. Via the RAS-ERK and PI3K-AKT pathways, IGF contributes to protein synthesis, cell growth, and cell proliferation [61]. IGF-treated DCs are unable to mature and secrete higher levels of TNF-α and IL-10, both of which are immune suppressors in the microenvironment of OC [62]. The differentiation of DCs into cDCs is negatively correlated with the high expression of the insulin-like growth factor type I receptor (IGF1R) in OC [63].

Scarlett et al. discovered that DC depletion at advanced stages significantly delays aggressive malignant progression. [59]. It is worth noting that mature myeloid DCs, due to their antitumor immune responses, have been used in clinical trials of DC vaccine therapy in OC [36,64].

Because DC functions can be influenced by their interactions with the proximal milieu, different DC locations may result in different functions [48]. Labidi-Galy et al. discovered that tumor pDCs released fewer pro-inflammatory cytokines than pDCs from ascites or peripheral blood in OC patients [48].

Furthermore, the DC performance can change depending on the stage of tumor development. In an OC mouse model, infiltrating DCs prevented tumor growth at an early stage. However, at an advanced stage, DCs become immunosuppressive in the TME, inhibiting anti-tumor T cell activity and DC depletion, significantly delaying the disease progression [59]. Similarly, Krempski et al. discovered that infiltrating DCs acquired a more immunosuppressive phenotype as the tumor progressed over time in a mouse model of OC, as shown by the increased PD-1 expression [65].

### 2.3. Myeloid-Derived Suppressor Cells

A diverse group of myeloid cells known as myeloid-derived suppressor cells (MDSCs) co-express the myeloid surface markers GR-1 and CD11b [66,67]. Three phenotypes of MDSCs are present: polymorphonuclear PMN-MDSC (also known as granulocytic MDSC), which are morphologically similar to neutrophils; M-MDSC, which morphologically and phenotypically resemble monocytes; and a limited subset of cells that are capable of producing myeloid colonies, such as myeloid progenitors and precursors [68].

The mechanism that induces MDSCs into the TME of OC is poorly understood. Many studies have proposed various pathways of MDSC production among tumor cell lines, emphasizing the relevance of each tumor type’s unique combination of inflammatory cytokines.

MDSCs are recruited to ascites of OC in a CXCR4-dependent way that needs cyclooxygenase-2 (COX2), the major enzyme in PGE2 synthesis. PGE2 is essential for both the production of CXCL12 and the expression of CXCR4 in MDSCs [69]. Furthermore, PGE2 and COX2 redirect a potential pathway for the recruitment and development of CD1a+ DCs to CD14 + CD33 + CD34+ monocytic MDSCs and induce the expression of MDSC-associated immunosuppressive factors [69,70].

Another potential mechanism for the induction of MDSCs in the TME of OC involves IL-6. In OC, IL-6 has a significant role in the development of tumor growth, angiogenesis, and tumor myeloid cell infiltration. IL-6 may act as a mediator in the differentiation of MDSCs. According to an experimental investigation by Wouters et al., IL-6 and its receptor IL-6R are opposing markers for the survival and infiltration of tumor-infiltrating myeloid cells. Longer disease-specific survival and lower mature myeloid cell infiltration were observed in tumors with a high IL-6R expression. On the other hand, tumors with a high epithelial IL-6 expression showed a significant tumor-infiltrating myeloid infiltration and were associated with a reduced survival time [71].

One of the primary ways MDSCs can restrict T cell differentiation is by producing Arg-1 [42]. Additionally, MDSCs release ROS and nitric oxide (NO), which nitrate signaling molecules downstream of the Fc gamma receptor IIIa (FcRIIIa) and decrease the activity of T cells and NK cells, respectively [68,72]. The direct inhibition of T cells occurs when superoxide and NO combine to form peroxynitrite (PNT), which nitrates T-cell receptors and limits the response of antigen−MHC complexes. PNT also inhibits chemokines that are unique to T cells, which reduces the combination of antigenic peptides to MHC and hinders T cell migration [73].

Furthermore, MDSCs induce the activation and proliferation of regulatory Treg cells [74]. It has also been proposed that MDSCs support both the conversion of naïve CD4+ T cells to translate into induce Treg cells (iTreg) and the expansion of natural Treg cells (nTreg) [75,76].

The expression of ADAM Metallopeptidase Domain 17 (ADAM17) on MDSC reduced the expression of CD62 ligand (CD62L) on CD4+ CD8+ T cells, limiting recirculation into the lymph nodes [77].

Last, but not least, multiple checkpoint molecules have been implicated in MDSC-mediated immunosuppression, with PD-L1 and cytotoxic T cell antigen 4 (CTLA-4) being key negative regulators of T cell activities [78,79,80]. CTLA-4 is expressed by Treg cells and primarily interacts with CD80/CD86 expressed by antigen-presenting cell (APC)-like DC. This interaction reduces APC-dependent T cell activation [81].

### 2.4. Regulatory T Cells (Treg)

Regulatory T cells are a diverse group of CD4+ T lymphocytes that express CD25+ and the transcription factor forkhead box P3 (FOXP3) [82]. Treg cells are critical for maintaining tolerance and preventing autoimmunity. However, developing cancers including OC, recruit Treg cells to establish local immunosuppression through a variety of mechanisms [82]. Tregs cells identified as CD4 + CD25+ FOXP3+ are abundant in OC patients’ peripheral blood, TME and ascites [83,84].

Numerous pathways contribute to the induction and differentiation of Tregs in the OC TME. The most important axis in the selective trafficking of Tregs cells to tumors is CCR4/CCL22 and CCL17 signaling. TAMs are the main source of CCL22, which has been shown to be substantially expressed in OC and ascites cells compared with normal ovaries at the mRNA level [83]. According to a study performed on 104 patients with OC, the accumulation of Tregs cells in tumors induced by CCL22 is associated with decreased survival rates and a higher risk of patient death. The migration of T cells was significantly reduced by CCL22 inhibition [83]. Treg cell infiltration is also reduced by blocking CCR5/CCL5, which were also involved in the trafficking of Tregs cells [85].

A significant Th17 response in the early stages of tumor development has been demonstrated to be replaced by a preponderance of Treg cells in the late stages, demonstrating that tumor progression can shape Treg participation in the local immune milieu [86]. Idoleamine 2,3-dioxygenase (IDO) accumulation in tumors causes the tryptophan catabolite kynurenine to be activated. Kynurenine then binds the aryl hydrocarbon receptor (AhR) on T cells, shifting the Th17/Tregs cell balance in favor of Treg cell generation. Moreover, kynurenine can bind AhR on TAMs, triggering the production of IDO in a feedback loop. Furthermore, tumor-associated DCs infiltrating in the OC TME influence Treg cell transformation. Dysfunctional DCs infiltrating the TME directly contribute to the induction of IL-10-producing Tregs [87]. Notably, hypoxia-induced CCL28 overexpression enhances Treg cell migration to the OC TME via a mechanism involving CCR10, which also leads to IL-10 secretion [88].

Cell-to-cell contact is achieved through the production of molecules such as CTLA-4 and the secretion of soluble mediators such as IL-10 and TGF-β, which limit immune activation [89]. The co-expression of CD39, CD73, and glycoprotein-A repetition predominate protein (GARP) on Treg cells may also result in enhanced suppression [90,91,92]. Interestingly, in both animal tumor models and human head and neck cancer, CD39 and CD73 have been linked to reduced local anti-tumor immunity [93,94,95]. When CD39 and CD73 are co-expressed, they can convert ATP to adenosine, inhibiting the metabolic activity of effector T cells, whereas GARP is implicated in relaying TGF-β inhibitory signals and increasing FOXP3 production [92,96].

TNF receptor 2 (TNFR2) expression on Treg cells has been characterized as a maximally suppressive Treg cell population [97,98,99]. TNF-induced induction of TNFR2 expression on Tregs occurs in an inflammatory environment [16]. TNF levels in OC patient serum and ascites are elevated, and existing research suggests the establishment of a “TNF network” that supports tumor progression [100]. Research has shown that TNFR2 + Tregs cells from ascites were the most potent suppressor T cell population [101]. They were abundantly present within the ascites and more suppressive than in the peripheral blood of OC patients [101].

### 2.5. Tumor Infiltrating Lymphocytes (TIL)

Tumor infiltrating lymphocytes (TILs) are lymphocytes that left the vasculature and have localized in the tumor stroma or intraepithelial. TILs are segregated into those that penetrate the tumor islet (intraepithelial) and those that reside in the peritumoral space (stromal). TILs are dominated by CD3+ cells, while the proportions of CD4+ and CD8+ cells vary between cancers. TILs have been proven to be a powerful factor that imposes tremendous pressure on many types of cancer, including OC [102].

TIL local infiltration denotes local immunity of immune cells surrounding or within the tumor [103]. The abundance of TILs in OC shows that this tumor has immunogenic potential. Moreover, numerous tumor-associated antigens (TAAs) can be identified by T cells, highlighting the potential benefits of immunotherapy in OC treatment approaches [89,104]. DCs are immune system sentinels that detect TAAs expressed on the surface of tumor cells and transport them to lymph nodes, where they activate T cell-mediated immunity, allowing T cells to bypass the immune system working station and attack the tumor [105]. These physiological processes occur in all OC patients with improved survival because normally generated lymphocytes are insufficient to attack and eliminate the tumor in advance [106,107].

### 2.6. NK Cells

NK cells are a subset of the innate lymphoid cells that play a significant role in safeguarding the organism from viral infection, early malignant transformation, and metastatic tumor dissemination. Based on CD16 and CD56 expression, they can be divided into two main populations: CD56bright/CD16− predominantly function to produce cytokines in the bloodstream and CD56dim/CD16+, which causes cytotoxicity in the tissues. NK cells develop from the common lymphoid progenitor in the bone marrow, are most closely related to T cells, and have similar capacities to release perforin and granzymes for direct target cell killing. Furthermore, they signal target cell death via the Fas and TNF-related apoptosis-inducing ligand (TRAIL) pathways and release pro-inflammatory cytokines such as IFN-γ and TNF [108].

The CD16 receptor, natural killer group 2D (NKG2D) receptor, and natural cytotoxicity receptors, such as NKp30, are the most significant cytotoxicity receptors that mediate NK cell-dependent immunosurveillance. Malignant diseases have been associated with defects in NK cell function, including impaired cytotoxicity and cytokine secretion, aberrant receptor and ligand expression, decreased NK cell number, and NK cell anergy [109]. Two primary mechanisms that block the function of NKG2D and NKp30 have been described in OC [110,111,112].

Ascites fluid from OC patients contains cancer cells that emit macrophage migration inhibitory factor (MIF), a chemokine that promotes the growth, migration, and metastasis of tumor cells. NKG2D is transcriptionally downregulated by MIF in NK cells, which reduces their capacity to eradicate tumor cells [110]. Second, elevated levels of soluble B7-H6, one of the ligands for the NKp30 receptor, suppress NK cell function in ascites. Increased soluble B7-H6 expression was linked to decreased NKp30 expression in tumor-associated NK cells and decreased NK cell activity [112]. Immune escape may be facilitated by reduced NK cell activities in the OC TME, and additional research into the underlying mechanism is necessary.

NK cells in the presence of IL-18 release the chemokines CCL3 and CCL4 that recruit immature DCs. Cross-talk between NKs and DCs increases the expression of CXCL9, CXCL10, and CCL5 on DCs, which can recruit effector CD8+ T cells to the TME [113]. NK cells are powerful inducers of the cDC chemoattractants XCL1 and CCL5. Tumor PGE2 synthesis could interfere with this mechanism and the ability of DC to secrete chemokines [114].

## 3. Tumor Progression

The transcoelomic route is the most common route of OC cell metastasis, and it is predicated on cancer cells detaching from the primary tumor and moving through the peritoneal fluid to the omentum, parietal, and visceral peritoneum, as well as direct extension of the tumor lesions to the adjacent organs. The additional route is via lymphatic vessels to pelvic and paraaortic lymph nodes. According to studies, about 70% of women with OC had peritoneal cavity metastases at the time of diagnosis [115,116]. OC tumor cells primarily colonize the omentum and form metastatic lesions, although micro-metastases frequently appear on the peritoneal surfaces. Microvasculature perfusion is inadequate in metastatic omentum and peritoneal tissues, according to immunohistochemical studies. Furthermore, the normal collagen network is disrupted in the metastatic omentum and peritoneum [117].

Presently, two hypotheses for the peritoneal metastasis model in OC have been proposed. The first hypothesis, which is related to the “seed and soil” concept, proposes that peritoneal metastasis of OC is induced by circulating tumor cells within the peritoneal cavity, which preferentially metastasize to the peritoneum through transcoelomic, hematogenous, or lymphatic routes [118]. The second hypothesis, known as the metaplasia hypothesis, argues that the OC metastatic omental sites are the consequence of a synchronized malignant transformation of the peritoneum or omentum due to the ovarian epithelium and omentum’s similar origin [115].

Although the etiology of ascites is unknown, preclinical and clinical studies have shown that the vascular endothelial growth factor (VEGF) is responsible for ascites accumulation, and that the obstruction of lymphatic vessels by cancer cells may also cause the accumulation of ascites. Ascitic fluid in most cases of OC is composed of malignant cells, a large number of immune cells, and a high level of lactate dehydrogenase (LDH) [119,120].

It should be emphasized that the conventional patterns of metastasis via the hematogenous pathway occur in OC, yet they are not the dominant way, and they are primarily responsible for distant metastases [13]. In clinical trials, inferior vena cava filters enhance the risk of hematogenous dissemination in OC by activating platelets and causing a proinflammatory response [121]. Moreover, circulating tumor cells detached from original lesions could be identified in OC patients upon early diagnosis [122].

The underlying mechanisms creating the strong OC tropism and complex interplay between tumor cells, immune cells, and platelets within the TME are unknown.

### 3.1. Tumor-Associated Macrophages

TAMs play a critical role in the transcoelomic dissemination of OC cells, their survival in ascitic fluid, and the formation of spheroids in malignant ascites. Furthermore, many spheroids in OC patients are heterogeneous, containing TAM-OC cells. TAMs have been found to promote spheroid formation and tumor growth in an ID8-bearing mouse model by secreting endothelial growth factor (EGF) [123].

To promote the interaction between TAMs and tumor tissues, EGF secreted by TAMs in the core of the spheroid upregulated the αMβ2 integrin on TAMs and intercellular cell adhesion molecule-1 (ICAM-1) on tumor cells. EGF influences ICAM-1 expression by inducing VEGF-C production, which promotes VEGF receptor 2 (VEGFR3) signaling in tumor cells and induces integrin/ICAM-1, which ultimately leads to tumor growth and migration [120,124]. In an animal model, inhibiting VEGF/VEGFR signaling or neutralizing ICAM-1 slowed spheroid and tumor growth [38].

Hypoxia is another essential component in the accumulation of TAMs. TAMs have been found in high-density hypoxic areas of tumors in many studies. According to Wen et al., increased 5-lipoxygenase (5-LOX) metabolites from OC cells under hypoxic conditions stimulate TAM migration and invasion by upregulating matrix metalloproteinase-7 (MMP-7) via the p38 pathway. The tumor-bearing mouse model demonstrates that inhibiting 5-LOX selectively reduces MMP-7 expression and the number of TAMs in tumor tissues [125].

Monocytes and macrophages obtained from OC patients had an increased number, a less differentiated phenotype, deficient cytotoxicity and phagocytic abilities, and impaired antitumor activities when compared with cells isolated from healthy donors [126]. An animal investigation showed that chemically depleting macrophages significantly reduced tumor dissemination and ascites build-up in OC-bearing mice [127].

In OC cells, the expression of MIF and extracellular matrix metalloproteinase inducer (EMMPRIN) upregulated MMP secretion by macrophages and overexpressed MMP promotes OC invasion and angiogenesis [128]. MIF inhibition in ovarian tumor-bearing mice decreases macrophage infiltration, as well as IL-6, IL-10, and TNF-α [129]. Furthermore, downregulated MIF in OC cells decreased the production of CCL2 and CCL22 in vitro [129]. CCL22, which is produced and increased by ovarian TAMs, can stimulate Treg cell trafficking to the tumor [83]. CCL18, an immunosuppressive chemokine expressed by TAMs, has been found in high levels in patients with OC. CCL18 levels in ascites and the serum of OC patients are elevated, which increases tumor migration and metastasis [130,131]. IFN-γ therapy can decrease CCL18 secretion and shift TAMs from being immunosuppressive to immunostimulatory [38].

Scavenger receptor-A (SR-A, CD204) expressed on TAMs and dependent on the presence of TNF-α was found to be involved in tumor cell invasion [33]. In vitro, SR-A-deficient macrophages diminish the invasiveness of OC cells. Tumor progression and metastasis are decreased in SR-A (-/-) mice when compared with the control group [132].

The crucial mechanism through which TAMs promote OC cell invasiveness is through the activation of nuclear factor κB (NFκB) activity. Numerous data from in vitro investigations on different cell lines clearly show that TNF-α is a crucial player in NFκB overexpression and activation in cancer cells. Blocking the NFκB signaling pathways in ovarian TAMs reduces M2 cytokines while increasing M1 IL-12 and NOS, thus inhibiting tumor cell invasion [133]. The effect of TNF-α on a tumor is determined by the dose and time. Prolonged low-dose exposure increases tumor progression, whereas a single high-dose exposure causes tumor regression. After interacting with its receptors, TNF-α increases the activity of OC cells, changes their shape, and promotes carcinogenesis and angiogenesis [133]. TNF-α expression is closely associated with the accumulation of IL-6 and CXCL12, which are implicated in an autocrine cytokine network, which was previously confirmed in OC [100]. In both a mouse model and human OC specimens, the “TNF network” played a paracrine function in angiogenesis, myeloid cell infiltration, and NOTCH signaling [100].

An elevated level of IL-6 and TGF-β in ascites and the serum of OC patients was associated with the generation of TAMs. STAT3 is a crucial point of junction for several carcinogenic pathways and is activated by TAMs IL-6, which is necessary for OC cell migration, motility, survival, and proliferation [134].

CXCR4 is found to be constitutively expressed in OC, while CXCL12 has been found to be highly concentrated in OC ascites. The CXCR4−CXCL12 pathway promotes invasion, the recruitment of immunosuppressive cells, and angiogenesis. Blocking the CXCR4−CXCL12 pathway improves tumor-bearing mice survival by decreasing the number of Treg cells and increasing the CD8/Treg ratio [135]. A study by Kajiyama et al. found that high levels of CXCL12 upregulated by TGF-β1 were accumulated in OC patient’s human peritoneal mesothelial cells (HPMCs), which served as scaffolding for the first step of peritoneal metastasis, enhancing crosstalk between tumor cells and HPMCs and promoting peritoneal metastasis [136].

TAMs may also be implicated in angiogenesis. The functional interaction of macrophages and endothelial cells is a hot topic in cancer and vascular biology. Co-cultivating OC cell lines with TAMs greatly boosts endothelial cell migration and tube formation, as well as the concentration of the pro-angiogenic cytokine IL-8 [137]. TAMs have also been linked to lymphangiogenesis in ovarian cancer. TAMs were reported to enhance lymphangiogenesis by stimulating lymphatic endothelial cell proliferation, migration, and capillary-like tube formation in a study of 108 ovarian tissue specimens. Its property may be enhanced by combining it with high-mobility group box protein 1 (HMGB1) [138].

To summarize, TAMs play a significant role in the course of OC disease. TAMs are a potent immunosuppressive driver within the TME. TAMs also promote metastasis and angiogenesis via several signaling pathways, which is especially important in OC, which is frequently diagnosed after the tumor has already metastasized.

### 3.2. Myeloid Derived Suppressor Cells

MDSCs not only suppress immune responses within the TME, but also promote cancer progression by stimulating tumor angiogenesis and enhancing tumor cell invasion and metastasis. MDSC under hypoxia-derived mediators, such as VEGF, basic fibroblast growth factor (FGF), and MMP-9, all play a crucial role in tumor angiogenesis and cancer cell invasion, regulating these processes [139]. Furthermore, new research suggests that MDSCs are involved in the establishment of epithelial-to-mesenchymal transition (EMT) or by forming “premetastatic niches” [140]. Crucially, data suggest that MDSCs promote “stemness”, which may be linked to resistance to conventional anticancer treatments such as chemotherapy or radiotherapy [141].

Additionally, by promoting miRNA101 expression and subsequently suppressing the co-repressor gene C-terminal binding protein-2 (CtBP2), MDSCs improve the stemness of OC cells. By specifically focusing on the stem-cell core genes, CtBP2 improves tumorigenic and metastatic potential while increasing the stemness of cancer cells [142].

### 3.3. Tumor Infiltrating Lymphocytes (TIL)

The absence of TILs in patients is linked to increased angiogenesis in OC, which creates a significant barrier to the infiltration of TILs. Overexpressed VEGF promotes endothelial cell proliferation, migration, and invasion in OC, which is associated with increased microvascular density [143]. Through the deregulation of ICAM-1 and vascular cell adhesion protein 1 (VCAM-1), VEGF-A can also lessen the adhesion between lymphocytes and tumor vascular endothelial cells, which in turn reduces TIL penetration [144]. Moreover, VEGF-A can promote the expression of FasL in endothelial cells in conjunction with IL-10 and PGE2, and increased FasL expression is linked to enabling the selective trafficking of more Tregs than CD8+ T cells [145]. Additionally, it has been demonstrated that VEGF inhibits CD8+ T cells by recruiting in MDSCs, and that the MDSCs produced by VEGF have stronger immunosuppressive properties. Anti-VEGF therapies successfully reduced the tumor development and ascites generation in mice models of OC [146].

### 3.4. Neutrophils

Neutrophils are a remarkably diverse population with both pro- and anti-tumor abilities, which is mostly due to phenotypic plasticity. Contrary to the notion that mature neutrophils exit the bone marrow as terminally differentiated cells, there are distinct subsets of neutrophils that differ in terms of their level of maturity and activation, as well as their pro- and anti-cancer properties. Neutrophil subsets with opposing roles have so far been identified in cancer patients’ circulation and primary tumors. The significance of neutrophils in cancer is debatable as they have seemingly contradicting qualities that might promote or inhibit tumor growth [147]. TAN (tumor-associated-neutrophils) can have anti-tumorigenic (N1) or pro-tumorigenic (N2) functions [148]. According to studies, TAN displays different activity patterns depending on the microenvironment. TGF-β induces neutrophils to acquire an N2 pro-tumoral phenotype, whereas IFN-β induces neutrophils to acquire an N1 phenotype, which is more anti-tumoral [148,149].

TGF-β inhibition enhanced the proinflammatory potential of TANs (N1), resulting in an increased expression of proinflammatory cytokines such as TNF-α and CCL3, cytotoxic CD8+ T lymphocyte activation, and direct killing of tumor cells dependent on ROS and the costimulatory molecule ICAM-1. [148]. N2 neutrophils have higher levels of CXCR4, VEGF, MMP-9, and arginase, which promote angiogenesis, immunological suppression, and tumorigenesis [150]. Moreover, according to in vivo research, subpopulations of TANs in early lung cancer could be polarized to an anti-tumor phenotype in response to low doses of GM-CSF and IFN-γ [151].

Neutrophils derived from bone marrow are generally found in low abundance in the omentum and peritoneal fluid, and act as the first line of defense in response to infections or tissue damage [152,153]. It has been reported that neutrophils mobilize into the abdominal cavity in response to peritoneal infection or injury via specialized vessels known as high endothelial venules (HEVs) in omental milky spots [154]. Lee et al. hypothesized that the establishment of the premetastatic omental niche in OC involves neutrophil influx at this site. It was discovered that recruiting neutrophils to the omentum is an important step preceding OC cell invasion of this site [155]. Moreover, Mishalian et al. proposed that neutrophil behavior varies depending on the stage of tumor development. They discovered that neutrophils were almost exclusively found at the tumor’s periphery during the initial stages of tumor development, and TANs were more cytotoxic toward tumor cells and generated higher levels of TNF-α, NO, and hydrogen peroxide (H2O2). At advanced stages of disease, neutrophils were found scattered among the tumor cells, and TAN properties, such as suppressing adaptive antitumor immunity, were down-regulated and acquired a more N2-pro-tumorigenic phenotype [156].

Neutrophils recruited into the TME are a source of cytokine and chemokine secretion that impacts innate and adaptive immunity. TANs release cytokines and chemokines, which have the ability to regulate both the activation and recruitment of other immune cells, as well as their own recruitment [157].

Neutrophils regulate immune responses using a variety of mechanisms, including the release of chemokines/cytokines, production of variety of antimicrobial molecules, neutrophil extracellular traps (NETs), recruitment of Treg cells, and PD-L1/PD-1 interactions [158,159,160]. Although some research implies that neutrophils have an antitumorogenic role due to T cell stimulatory activities, the majority of studies thus far indicate that neutrophils mostly have immunosuppressive functions.

In the case of OC, neutrophils work in tandem with TNF-/IL-17 signaling to promote tumor development and enhance premetastatic niche creation via NETs [161]. Furthermore, neutrophils developed inhibitory behavior in response to ascites supernatants from advanced OC patients. Additionally, Emmons et al. demonstrated the ability of TANs (N2) to adhere to T cells and cause trogocytosis of T-cell membranes, resulting in T-cell immunoparalysis characterized by an impaired nuclear factor of activated T cell (NFAT) translocation [162].

### 3.5. Platelets

Activated platelets play a variety of roles in tumor metastasis progression, including facilitating tumor-cell EMT. They become less adherent, less polar, more mesenchymal, and have increased mobility. EMT is characterized by a decreased expression of E-cadherin following the increased expression of p38 and related pathways. It has been shown that E-cadherin is down-regulated by epidermal growth factor receptor (EGFR) activated by OC cells, and that cells with a low E-cadherin expression are particularly invasive [163].

The role of platelets in EMT has been connected to many factors derived from either platelets or tumors to induce platelet activation. Podoplanin (PDPN) is a glycoprotein that binds to C-type lectin-like receptor 2 (CLEC-2) on platelets, resulting in platelet activation. PDPN-positive cancer cell lines stimulate platelet activation, particularly platelet TGFβ-expression, which induces cancer cells to undergo EMT. TGF-β inhibition, on the other hand, strongly reduces PDPN-induced EMT and metastasis in mice injected with tumor cells, implying that podoplanin causes tumor metastasis by boosting platelet-derived TGF-β [164].

Among the platelet associated metastasis mediators, TGF-β plays an essential role. It has been reported that in OC, there is a significant correlation between elevated platelets counts with a higher incidence of intraperitoneal dissemination and higher TGF-β [165]. Furthermore, platelets promote EMT and the invasiveness of OC cells by activating the TGF-β/Smad pathway. All of these impacts were reversed through the use of antiplatelet agent A83-01 (a selective inhibitor of TβRI), which inhibits the activation of the Smad pathway and, as a result, blocked the pro-metastatic effects and reversed the EMT in tumor-bearing OC cells in mice [165].

Additionally, the contribution of platelets to metastasis is influenced by autotaxin (ATX), which regulates lysophosphatidic acid levels (LPA) [166]. LPA acts as a bridge between cancer cells and platelets in the process of tumor invasion and metastasis. LPA is prevalent in the malignant ascites of OC patients [167,168]. In contrast with normal ovarian epithelial cells, which do not produce LPA at levels sufficient to stimulate aberrant receptors, the constitutive production of LPA by both OC cells and peritoneal mesothelial cells accelerates the spread of cancer cell and the synthesis of IL-6 and IL-8 [169,170]. CD97, a member of the epidermal growth factor family that is activated in various types of cancer, activates the signaling pathway via the LPA receptor [171].

There is mounting evidence to support the notion that platelets shape the metastatic milieu in the setting of the early metastatic niche. This was demonstrated in a lung cancer mouse model, where tumor-aggregated platelets promoted the formation of metastatic sites by producing cytokines that attract granulocytes CXCL-5 and CXCL-7 [172]. Platelet-derived chemoattractants, such as C-X-C motif ligands, particularly trigger the deployment of granulocytes to early metastatic niches rather than monocytes, lymphocytes, or NK cells [172]. Orellana et al. revealed that platelets have a chemotactic effect on OC cells, with the subsequent phenotypic change favoring a mesenchymal phenotype, with an enhanced expression of the tissue factor (TF) [173]. Platelets thus not only give survival signals for tumor cells, but also recruit host cells to disseminate tumor foci [173].

In order for cancer cells to escape detachment-induced apoptosis (often known as anoikis), platelet−cancer-cell interaction is required. Experimental findings implicate a crucial role for platelets in inducing anoikis resistance and in the metastatic spread of cancer cells intraperitoneally and hematogenously by inducing a Yes-Associated-Protein 1(YAP1)-dependent transcriptional program in detached cancer cells, which promotes cell survival and metastasis [174]. Furthermore, the results suggest that reducing blood platelet counts or interfering with YAP1 signaling might be an important approach to limit OC metastasis [174]. Additionally, recent research by Rodriguez-Martinez et al. has strengthened the notion that platelet interaction may cause tumor cells to develop aggressive phenotypes such as EMT, stem-like phenotypes, and high rates of proliferation [175]. The findings also showed that platelets educate tumor cells through the very effective transfer of lipids, proteins, and RNA via various mechanisms [175].

Cancer cells are exposed to significant challenges such as shear forces during circulation before reaching the distant site [176]. Platelets support cancer spread by shielding cancer cells from shear pressures induced by blood flow. The co-incubation of A2780 OC cells with human platelets significantly reduces the cancer cells’ release of LDH, a marker for shear-induced membrane damage [176]. Platelets can protect OC cells against shear-induced damage. The disruption of platelet−cancer-cell cross talk could increase the shear- stress-induced destruction of cancer cells in vivo [176].

Immune attack, in which NK cells play a crucial role in the immune system, present another significant threat to metastasizing cells in circulation [177]. Through the formation of aggregates on the tumor cell surface, platelets protect tumor cells against NK cell lysis [177]. Both sterically and by preventing NK cells from performing their cytolytic function, platelets prevent NK cells from adhering to tumor cells. Platelet activation leads to the expression of the TNF receptor superfamily member glucocorticoid-induced TNF-related ligand (GITRL) in parallel with the α-granular activation marker P-selectin on their surface membrane [178]. GITRL binds to its receptor on the NK cell membrane and suppresses the latter’s cytotoxic properties by impairing its lytic activity and IFN-γ secretion [178]. Activated platelets also contain a plethora of soluble substances that suppress NK cells. TGF-β released during tumor-cell-induced platelet aggregation (TCIPA) has been shown to downregulate theNKG2D immunoreceptor, limiting lytic activity and IFN-γ secretion [179]. It has also been proposed that the transfer of platelet-derived MHC-I onto the surface of tumor cells during aggregation may restrict the immune system’s NK-mediated attack against the developing metastatic niche [179]. Apart from NK cells, platelets interact with other immune cells such as macrophages and T-cells, and their impact on them may contribute to the formation of an immunosuppressive milieu. The micrometastatic niche, for example, is rich in platelet and tumor-cell-derived TGF-β, which suppresses both CD4+ and CD8+ T-cell activity.

Furthermore, platelets have been linked to neutrophil activation and the formation of neutrophil NETs, a process known as NETosis. The relationship between platelets and neutrophils is bidirectional as platelet TLR4 promotes NETosis and extracellular DNA from NETs induces platelet activation [180]. It also contributes to cancer-associated thrombosis, which OC patients are at a higher risk of developing [181,182,183].

According to the research, either by the direct paracrine effect on megakaryocytes or through their ability to generate and activate platelets, cancer cells can affect platelet counts and physiological activation states. OC cells can directly activate platelets through contact or indirectly through the release of agonist-like substances that contribute to the stimulation of megakaryopoiesis and, consequently, thrombopoiesis in cancer patients [184,185,186]. Platelets are activated via key pathways such as thromboxane (TX)-A2, glycoprotein (GP)-Ib-IX, adenosine diphosphate (ADP), and GPIIb/IIIa [187].

Additionally, through participation in the coagulation pathway, cancer cells might indirectly activate platelets. TF, which may be generated and secreted by macrophages and endothelial cells as well as OC themselves, is released in great quantities by cancer cells and is a key factor in the procoagulant ability of tumors [188,189,190]. Furthermore, cancer cells have the capacity to release TF-rich procoagulant microparticles (MPs) that cause the production of thrombin [191]. Thrombin secretion is one of TCIPA’s most crucial processes [192,193]. In addition to activating the receptor PAR on the surface of platelets and the coagulation factors V, VIII, XI, and XII, thrombin transforms fibrinogen to fibrin [194].

Platelets have long been known to play an active role in angiogenesis, from the initial stages of vasculogenesis through the advanced stages.

While it has been demonstrated that platelets triggered by VEGF assist in the extravasation and metastasis of tumor cells, platelet-derived VEGF also enhances angiogenesis by encouraging the recruitment of further endothelial cells in the TME in OC [195]. In vitro investigations have shown that exposing OC cell lines to activated platelets significantly increases VEGF production in a cellular medium [196]. The endothelial markers VEGF and CD31 were discovered to co-localize with platelets in murine models of OC, while co-culture of human OC cell lines with platelets increased the secretion of many pro-angiogenic factors [197,198]. Surprisingly, this preclinical evidence reveals that metformin may inhibit platelet pro-angiogenic activity in OC [198]. Platelets may also play a role in tumor growth once anti-angiogenic therapy is discontinued; discontinuation of anti-VEGF medications is associated with accelerated tumor growth and simultaneous tumor platelet infiltration, while platelet depletion mitigates these effects in vivo [199].

## 4. Prognostic Significance of Immune Cell and Platelets in Ovarian Cancer

Many aspects of the immunological response to OC have predictive value. The presence of various immune cells and platelets infiltrating or interacting with the tumor has been linked to better and worse disease prognoses, indicating pro- or anti-tumor activities. Investigations on diverse tumor tissues have revealed significant infiltration of immune cells and platelets into the intratumoral and peritumoral areas. The presence of these cells is linked to tumor responses to a plethora of cytokines and chemokines produced and released by tumor cells. Its detection in OC allows for better clinical outcome prediction than other histological markers, indicating that the immunological profile can be useful in alternate forms of treatment, such as immunotherapy.

### 4.1. Tumor-Associated Macrophages

Research on the presence of TAMs in OC shows that the number of TAMs in malignant ovarian tumors is significantly higher than in benign and borderline tumors [200,201].

High numbers of M2-like TAMs in primary and metastatic OC of all histologies are associated with a lower sensitivity to treatment and a poor prognosis.

M1-like TAMs are a positive prognostic factor in women with EOC, owing to their ability to promote robust inflammatory responses that limit disease progression, despite the fact that their presence is significantly reduced in the TME of patients with advanced OC (Table 1).

Retrospective research of patients with advanced OC examined the expression of TAMs in tumor tissues using CD68 and CD163 as M1 and M2 macrophage markers, respectively. When comparing high vs. low CD163 (M2 TAMs), there was a substantial difference in progression-free (PFS) and overall survival (OS), with the low CD163+ groups significantly exceeding the high CD163+ groups [202]. Yafei et al. investigated the predictive value of CD68+ and CD163+ positive macrophages in 42 OC patients at various stages of disease. Immunohistochemical research revealed that a high proportion of CD163+ (M2 phenotype) in the total CD68+ macrophages predicted a poor prognosis [203].

A meta-analysis of 794 OC patients was performed to determine the relationship between the TAMs phenotype and clinical outcomes [204]. The infiltration of tumor tissue with CD163+ TAMs was associated with a poor prognosis, whereas a high M1-to-M2 macrophage ratio predicted a better prognosis for both OS and PFS. Another study on 112 patients with advanced OC found that a high M1/M2 ratio of TAMs in tumor specimens was associated better disease outcome [205].

Another M2-related marker was linked to a worse outcome in OC. While absolute CD206+ cell counts were not predictive, a high CD206/CD68 ratio was substantially associated with a worse PFS and worse OS [206]. Research on M1 (HLA-DR and iNOS) and M2-polarization (CD163 and VEGF) markers in OC patients found that a greater M1/M2 ratio was associated with improved patient survival [40,205]. Zhang et al. measured the M1/M2 ratio in the tumor and the stroma, and discovered that only the M1/M2 ratio of overall tumor macrophages or macrophages present intratumorally were prognostic, while the M1/M2 ratio in the tumor stroma was not, indicating that macrophages infiltrating tumor cells may play a more important role in tumor progression [205]. Furthermore, B7-H4 expression on the surface of TAMs, but not in ovarian tumor cells, was associated with decreased survival, and the number of B7-H4+ macrophages was significantly increased in advanced stages of disease [43].

**Table 1:** Characteristics of studies on tumor-associated macrophages and ovarian cancer prognostic significance.

Author/Year	Histology	Stage	Method	Impact	Findings
Tan et al., 2021 [207]	n.a	n.a	Meta-analysis	Detrimental	High density of M2 TAMs negatively correlated with OS.
Sue-A-Quan et al., 2021 [208]	CCOC	I-III	IHC	Detrimental	High density of CD68+ TAMs was associated with shorter DFS, RFS and OS.
Macciò et al., 2020 [209]	EOC	IIIC-IV	FC	Beneficial	High density of M1 TAMs, and high M1/M2 ratio correlated with longer OS, PFS and PFI.
Badmann et al., 2020 [210]	EOC	I-IV	IHC, IF	Detrimental	Infiltration of MDR1+ CD163 + CD68+ M2 TAMs was associated with poor prognosis.
Yuan et al., 2017 [204]	n.a	I-IV	Meta-analysis	Detrimental	Higher M1/M2 TAMs was associated with a favorable OS and PFS. High density of CD163+ M2 TAMs correlated with poor disease outcome.
Yin et al., 2016 [123]	EOC	III-IV	IHC	Detrimental	High density of EGF-secreting M2-like TAMs correlated with poor disease outcome.
Zhang et al., 2014 [205]	EOC	I-IV	IHC, IF, FC	Beneficial	High M1/M2 TAMs ratio correlated with improved prognosis.
Reinartz et al., 2014 [31]	HGSOC	I-IV	FC	Detrimental	High density of CD163+ TAMs correlated with poor RFS and OS.
He et al., 2013 [40]	EOC	II-IV	IHC, IF	Detrimental	High density of M2 TAMs negatively correlated with OS.

EOC, various subtypes; CCOC, clear cell carcinoma; HGSOC, high-grade serous ovarian cancer; OS, overall survival; PFS, progression-free survival; PFI, platinum-free interval; RFS, relapse-free survival; DFS, disease-free survival, IHC, immunohistochemistry; IF, immunofluorescence; FC, flow cytometry; n.a, not available.

### 4.2. Dendritic Cells

Recently, studies have shown that DCs may infiltrate OC tumors and have a beneficial or adverse prognostic impact on patients, depending on the subpopulation (Table 2). According to research carried out on HGSOC cancer patients, the presence of mature, DC-LAMP+ DCs in TMA was correlated with improved OS. Furthermore, it should be noted that most mature DC-LAMP+ DCs are found in the tumor stroma and are linked to tertiary lymphoid structures (TLSs) rather than being in close proximity to malignant cells [211,212]. However, a higher density of mature DCs in the ovarian TME is associated with cytotoxic activity and Th1 polarization biomarkers, both of which are promising indicators in patients with OC [211,212].

Mastelic-Gavillet et al.’s investigation showed that a high expression of CLEC9A (cDC1s marker) in tissues of OC patients was associated with better OS. However, cDC1s are quantitatively and qualitatively impaired in patients with OC [213].

A high density of pDC in TME is often connected to immunosuppression and a worse outcome in OC. In the cohort studies of 44 patients, the accumulation of CD4+ BDCA2+ CD123+ pDC in TME, but not in ascites, was an independent prognostic factor associated with early relapse [48]. Notably, while pDC were the most abundant DC subgroup in TME and ascites, they were greatly diminished in the peripheral blood [48]. Similar results were obtained in immunohistochemical research performed on a cohort of 99 patients, where the presence of tissue-associated pCDs was correlated with PFS (14.6 months compared with 26.2 months in the absence of tissue-associated pDCs) [214]. According to these findings, pDC are mostly recruited into tumors where they exhibit a partially mature phenotype that would indicate in situ activation [48,214].

These findings indicate that while DC immunotherapies have considerable promise for the treatment of OC, they should be performed in conjunction with strategies for avoiding the quantitative and functional defects of DCs [215]. It has been demonstrated that treatment with CD40 and TLR3 agonists may transform tumor-associated DC into DC capable of stimulating anti-tumor activation of T-cells [216,217]. Moreover, it should target the subset that plays the most significant role in eliciting an anti-tumor response.

**Table 2:** Characteristics of studies on dendritic cells and ovarian cancer prognostic significance.

Author/Year	Histology	Stage	Method	Impact	Findings
Hensler et al., 2020 [211]	HGSOC	III-IV	IHC	Beneficial	High density of mature DC-LAMP+ DCs in peritoneal metastasis correlated with improved disease outcome.
Mastelic-Gavillet et al., 2020 [213]	n.a	I-IV	FC	Beneficial	High expression of CLEC9A (cDC1s marker) in tissues was associated with better OS.
Truxova et al., 2018 [212]	HGSOC	I-IV	IHC	Beneficial	High density of tumor-infiltrating DC-LAMP+ mDCs correlated with cytotoxic activity and favorable OS.
Zhang et al., 2015 [218]	EOC	I-IV	IHC	Beneficial	High density of CD1a+ mDCs correlated with improve OS.
Labidi-Galy et al., 2012 [214]	n.a	n.a	IHC	Detrimental	CD4 + BDCA2 + CD123+ pDC in tumors correlated with poor disease outcome.
Conrad et al., 2012 [60]	EOC	II-IV	IHC, FC	Detrimental	High density of HLA-DR + CD123+ pDCs and ICOS+ FOXP3+ Treg cells correlated with poor disease outcome.

EOC various subtypes; HGSOC, high-grade serous ovarian cancer; OS, overall survival; IHC, immunohistochemistry; FC, flow cytometry; n.a, not available.

### 4.3. Myeloid-Derived Suppressor Cells

Correlative studies in humans linking the number and phenotype of MDSC with a poor prognosis, as well as animal studies, have revealed the clinical importance of MDSCs. A higher MDSC frequency in the tumor tissue, peripheral blood, or ascites is linked with a lower OS or relapse-free survival (Table 3). Mouse model studies have shown that MDSC accumulation during tumor formation and the ablation of MDSCs resulted in improved survival [219]. Lee et al. discovered that women with germline BRCA1- and BRCA2-mutation-associated OC have fewer circulating immunosuppressive immune cells compared with those with BRCA wild-type hereditary BRCA1 OC, which is thought to respond better to platinum-based chemotherapy than BRCA wild-type OC and has fewer circulating MDSCs and greater CD8+ T cells in peripheral blood mononuclear cells (PBMC) than BRCA wild-type OC [220]. Additionally, Li et al. found that metformin treatment in diabetic OC patients was related to lower circulating MDSCs, an increase in circulating CD8+ T cells, and a longer survival [221].

MDSCs decreased the antitumor immunity by using a variety of inflammatory mediators. Okła et al. performed a comprehensive analysis of the MDSC subpopulation and immunosuppressive factors, finding that high levels of M-MDSCs were associated with an advanced stage and high grade of OC and were significantly increased in blood-circulating immunosuppressive factors (Arg, IDO, and IL-10) compared with the healthy group. Furthermore, a decreased level of M-MDSCs was strongly correlated with prolonged survival in OC patients [222]. Wu et al. revealed that IL-6 and IL-10 from ascites increased the recruitment/production of M-MDSC in the peripheral blood and ascitic of OC patients and high levels of M-MDSC were correlated with a poor prognosis [223]. Additionally, the M-MDSC/DC cell ratio is an independent predictive factor for EOC survival and a high ratio is correlated with poor OS [224]. All of these findings suggest that the M-MDSCs subpopulation may have the highest clinical significance in MDSC populations.

Although all of these studies have demonstrated that MDSC has a predictive value in OC, they have significant drawbacks, such as a small study group, the use of variety of MDSC surface markers, and most studies being conducted in a single institution.

**Table 3:** Characteristics of studies on MDSC and ovarian cancer prognostic significance.

Author/Year	Histology	Stage	Method	Impact	Findings
Okła et al., 2020 [225]	EOC	I-IV	IHC	None	High density of PD-L1+ MDSCs was not associated. with disease outcome.
Komura et al. 2020 [141]	n.a	n.a	IHC, FC	Detrimental	MDSC increases the stem cell-like properties and tumor PD-L1 expression by PGE2 production.
Okła et al., 2019 [222]	EOC	I-IV	FC	Detrimental	High density of M-MDSCs in peritoneal fluid correlated with poor disease outcome.
Lee et al., 2019 [220]	HGSOC, OCCC	III-IV	FC	Beneficial	Patients with gBRCAm mutation correlated with low level of MDSC in peripheral blood and better disease outcome.
Li et al., 2018 [221]	EOC	I-IV	FC	Detrimental	Metformin inhibition of CD39+/CD73+ MDSCs improve antitumor T-cell immunity.
Taki et al., 2018 [226]	HGSOC	III-IV	IHC, FC	Detrimental	High MDSC infiltration correlated with high CXCL1/2 level and short OS.
Santegoets et al., 2018 [224]	EOC	n.a	FC	Detrimental	High density of M-MDSC and low M-MDSC/DC ratio are associated with poor disease outcome.
Horikawa et al., 2017 [146]	HGSOC	III-IV	IHC, FC	Detrimental	High VEGF expression induced MDSCs and correlated with poor prognosis.
Cui et al., 2013 [142]	HGSOC	I-IV	IHC, FC	Detrimental	High density of MDSC, significantly correlated with poor disease outcome.
Obermajer et al., 2011 [135]	n.a	III-IV	FC	Detrimental	High CXCL12 levels correlated. with accumulation of MDSCs in malignant ascites and poor disease outcome.

EOC various subtypes; CCOC, clear cell carcinoma; HGSOC, high-grade serous ovarian cancer; OS, overall survival; IHC, immunohistochemistry; FC, flow cytometry; n.a, not available; gBRCAm, germline mutation.

### 4.4. Lymphocytes

There has been extensive research in the last 20 year regarding TILs and their potential as predictive biomarkers in patients with OC (Table 4). Several publications indicate that a higher level of intraepithelial CD3+, CD8+, and CD103+ cells is associated with a more favorable clinical outcome [227,228,229].

Importantly, a study of 186 OC patients with an advanced stage of disease (FIGO III/IV) found that patients with CD3+ TILs had a 5-year OS rate of 38.0%, while patients without detectable CD3+ TILs had a 5-year overall survival rate of only 4.5%. Moreover, complete responses (CR) occurred in 73.9% of patients with CD3+ TILs compared with 11.9% of patients without TILs who underwent surgical debulking and platinum-based chemotherapy [227].

According to Hamanishi et al., study patients with CD8+ TILs had longer PFS and OS [230]. Similar results were found by Sato et al.; patients with higher percentages of CD8+ TILs demonstrated improved survival compared with patients with lower percentages (55 months vs. 26 months) [227]. Nonetheless, the groups with high vs. low CD8+/CD4+ TILs ratios had a median survival of 74 and 25 months, respectively. These findings indicate that CD4+ TILs influenced the beneficial outcomes of CD8+ TILs [227]. In retrospective study of 500 OC patients, intraepithelial CD8+ TILs were correlated with improved disease-specific survival (DSS) from serous ovarian carcinomas, but not in endometrioid or clear cell carcinomas [231].

A meta-analysis of 19 studies comprising 6004 patients with HGSOC provided evidence of CD3+, CD4+, CD8+, and CD103+ TILs positively correlated with OS and PFS [232]. Furthermore, patients with primary or metastatic lesions with a high density of CD8+ TILs and a high CD8+/FOXP3+ cell ratio had a better DSS [233].

There are inconsistent data on Treg FoxP3+ cells, with the majority of studies supporting the association between Treg cells and a poor prognosis, while some studies revealed no association or even a beneficial effect on OS. Curiel et al. divided patients based on their numbers of CD4 + CD25 + FOXP3+ Treg cells (high, medium, and low), and found that Treg cells in tumor sites were associated with poor survival and a significant risk of mortality. Patients in the high Treg cell group experienced a 25.1-fold higher death hazard compared with those in the low Treg cell group [83]. Similarly, Herman et al. found that FoxP3+ T cells located within lymphoid aggregates surrounding the tumor were strongly associated with a reduced survival time [234]. Additionally, a high Treg/Th17 ratio was an independent poor prognostic factor for OS in OC patients [235]. In apparent contrast with other reports, Milne et al. revealed that the presence of intraepithelial FoxP3+ T cells was associated with increased DSS [236]. However, in a big meta-analysis across diverse types of cancer, including OC, it was not concluded that FoxP3+ Tregs cells in OC patients are a significant prognostic indicator of survival [237].

Furthermore, increased CD20+ TIL levels are associated with improved survival in OC patients [236,238]. Intratumor infiltration of CD27- atypical memory B cells, together with CD8+ T cells in HGSOC, is associated with a better prognosis [239]. Kroeger et al. discovered that a high infiltration of CD8+ T cells, CD20+ B cells, and plasma cells in OC tumors was associated with the presence of TLS in the TME and improved patient survival. Importantly, tumors containing CD8+ TILs only had an improved prognosis when identified in conjunction with CD20+ B cells, CD4+ T cells, and plasma cells, indicating cooperation between those subsets and their involvement in supporting antitumor immunity [240]. However, another study showed that a high expression of CD138+ plasma cells was correlated with a significantly reduced OS and OC-specific survival [241].

Furthermore, PD-1 and PD-L1 can be regarded as independent prognostic factors in OC [238]. Although the earliest findings on PD-L1 in OC suggested that PD-L1 expression on tumor cells was associated with a poor prognosis, some recent studies have revealed no association or even an improved outcome [230,242,243,244].

**Table 4:** Characteristics of studies on lymphocytes and ovarian cancer prognostic significance.

Author/Year	Histology	Stage	Method	Impact	Findings
Hao et al., 2020 [232]	HGSOC	I-IV	Meta-analyses	Beneficial	CD3+, CD4+, CD8+, and CD103+ TILs positively correlated with PFS, OS.
Henriksen et al., 2020 [245]	HGSOC	I-IV	IHC	Beneficial	High density of CD8+ TILs and PD-L1 expression correlated with favorable prognostic outcome.
Zhu et al., 2020 [246]	EOC	II-IV	Retrospective analysis	Beneficial	High density of CD38+ was associated with longer DFS and increased OS.
Fucikova et al., 2019 [247]	HGSOC	I-IV	IHC, FC	Detrimental	High density of PD-1+ TIM-3+ CD8+ T cells correlated with poor disease outcome.
Truxova et al., 2018 [212]	HGSOC	I-IV	IHC	Beneficial	High density of CD8+ T cells and CD20+ B cells correlated with improve survival.
Zhou et al., 2018 [235]	EOC	III-IV	FC, miRNA microarray	Detrimental	High Treg cells/Th17 cells ratio, derived by TAMs correlated with disease progression and metastasis potential.
Li et al., 2017 [248]	EOC	III-IV	Meta-analysis	Beneficial	Intraepithelial CD3+ and CD8+ CD103 + TILs correlated with improved survival.
Wang et al., 2017 [249]	HGSOC	I-IV	IHC	Beneficial	CD3+ or CD8+ TILs were positively correlated with longer OS.
Montfort et al., 2017 [250]	HGSOC	III-IV	IHC, FC	Beneficial	High density of memory 20+ B cells in omental metastasis correlated with cytolytic favorable disease outcome.
Darb-Esfahan et al., 2016 [251]	HGSOC	I-IV	IHC	Beneficial	High density of CD3+ T cells and high expression of PD-L1 correlated with PFS and OS.
Santoiemma et al., 2016 [238]	EOC	I-IV	IHC	Beneficial	High density of CD8+ T cells and CD20+ B cells TILs positively correlated with OS
Lundgren et al., 2016 [241]	EOC	I-IV	IHC	Detrimental	High density of CD138+ plasma cells correlated with poor OS.
Kroeger et al., 2016 [240]	HGSOC	I-IV	IHC, FC	Beneficial	TLS with high density of CD8+, CD4+ T cell, CD20+ B cells and plasma cells (CD20− CD38, CD138, and CD79a) correlated improve disease outcome.
Zhang et al., 2015 [218]	EOC	I-IV	IHC	Beneficial	High density of CD45RO+ T cells correlated with higher survival rates
Shang et al., 2015 [237]	n.a	I-IV	Meta-analysis	None	FoxP3 + Tregs cells did not correlated with OS.
Knutson et al., 2015 [252]	EOC	I-IV	IF	Beneficial	High CD8+ T cells and CD4+/Treg cells ratio was associated with improved OS.
Webb et al., 2014 [253]	EOC	I-III	IHC, FC	Beneficial	Intraepithelial CD103+, CD8+ TILs correlated with improved survival.
Hermans et al., 2014 [234]	EOC	I-IV	IHC	Beneficial/ Detrimental	CD8+ TILs positively correlated with OS. Treg cells lymphoid aggregates correlated with reduce OS.
Iglesia et al., 2014 [254]	n.a	n.a	Retrospective analysis	Beneficial	BCR gene segments correlated with improved prognosis.
Bachmayr-Heyda et al., 2013 [255]	EOC	II-IV	IHC	Beneficial	High densities of CD8+ TILs correlated with better OS.
Hwang et al., 2012 [256]	n.a	n.a	Meta-analyses	Beneficial	Intraepithelial CD3+ CD8+ TILs correlated with a improve survival.
Nielsen et al., 2012 [239]	HGSOC	II-IV	IHC, FC	Beneficial	The presence of both CD20+ and CD8+ TIL correlated with increased patient survival. Additionally, CD27-CD20+ memory B cells correlated with cytolytic immune response and favorable prognosis.
Callahan et al., 2010 [257]	EOC	IIIB-IV	IHC	Beneficial	Tumor cell expression of HLA-CD8 + TILs correlated with improved survival.
Barnett et al., 2010 [258]	EOC	I-IV	IHC, IF	Beneficial	CD8+ TILs positively correlated with improved disease outcome.
Leffers et al., 2009 [233]	EOC	I–IV	IHC	Beneficial	High densities of CD8+, CD45R0+, Treg and a high ratio CD8+/FoxP3+ Treg correlated with DSS PFS, OS.
Milne et al., 2009 [236]	HGSOC	I-IV	IHC	Beneficial	High density of CD3+, CD8+, Treg and CD20+ B cells correlated with improved disease outcome
Stumpf et al., 2009 [259]	SOC	III	IHC	Beneficial	Intraepithelial CD3+ CD8+ correlated with improved DFS and OS
Hamanishi et al., 2007 [230]	EOC	I-IV	IHC	Detrimental	Intraepithelial CD8+ TILs count was poor prognostic factor for PFS and OS.
Kryczek et al., 2007 [43]	EOC	I-IV	IF	Detrimental	Treg cells and TAMs B7-H4, correlated with poor disease outcome.
Sato et al., 2005 [227]	EOC	I-IV	IHC	Beneficial	High density of intraepithelial CD8+ T cells and high CD8+/Treg cells ratio correlated with favorable outcome.
Curiel et al., 2004 [83]	n.a	I-IV	IF, FC	Detrimental	Treg cells in tumor and malignant ascites were associated with poor survival.

EOC various subtypes; HGSOC, high-grade serous ovarian cancer; SOC, serous ovarian cancer; OS, overall survival; DFS, disease-free survival; DSS, disease specific survival; PFS, progression-free survival; IHC, immunohistochemistry; IF, immunofluorescence; FC, flow cytometry; CD45R0+, memory T-lymphocytes; TLS, tertiary lymphoid structures; n.a, not available.

### 4.5. NK Cells

Despite the fact that only a few studies have been conducted to assess the prognostic significance of NK cells in OC, most have presented promising outcomes (Table 5). Immunohistochemical research revealed that a high level of CD56+ NK cells was correlated with a favorable prognostic impact; the OS was 45 months in patients with a high level of CD57+ NK cells compared with a 29-month OS in patients with a low level of CD56+ NK cells [245]. According to similar findings, in ascites of OC patients, a higher percentage of CD56+ NK cells was associated with a better PFS, and their immunity was boosted by IL-15, which could be a promising direction for new immunotherapy [260]. Another study using anti-CD103+ antibodies discovered that CD103 was highly expressed by intraepithelial CD8+ T cells and in also some patients by NK cells TILs in human ovarian tumors. Significantly, CD103 + TILs comprising intraepithelial, CD8+ T cells, and NK cells were strongly associated with survival in HGSOC patients, indicating that CD103 represents a clinically beneficial TIL subset that merits further investigation [253].

The prognostic value of the blood NK cell count and intratumoral NK cell density in OC could be a promising therapeutic therapy for OC patients.

### 4.6. Neutrophils

In recent years, the role of neutrophils in OC has attracted more attention, and theneutrophilia and neutrophil-to-lymphocytes ratio (NLR) has been associated with detrimental outcome in OC patients (Table 6). A limited number of studies have investigated the prognostic role of neutrophilia in OC. Komura et al. suggested that neutrophilia is a poor prognostic factor, but is inferior to NLR [262]. However, according to two earlier findings, neutrophilia is not a prognostic factor in relation to the disease outcome [263,264].

Multiple studies have concentrated on the value of the inflammatory composite marker NLR in which most reported a strong predictive value for predicting the survival of patients with OC. In a retrospective analysis of 315 newly diagnosed EOC patients undergoing platinum-taxane chemotherapy, it was found that a high pre-treatment NLR count was an independent negative prognostic factor for PFS, but not OS [265]. A similar observation was reported by Wang et al., who recognized a preoperative high NLR count as a prognostic factor for both OS and PFS [266]. It also was suggested that a high preoperative NLR ratio was associated with a greater risk of 30-day postoperative morbidity and poor OS [267]. Notably, a similar observation was reported by Nakamura et al., correlating a high NLR count with a higher mortality within 100 days of unsuccessful first-line chemotherapy [268].

Furthermore, preoperative NLR combined with CA125 was shown to be useful for the early detection of OC and to be able to predict the pathological diagnosis of adnexal masses [269,270]. Additionally, a high baseline NLR ratio was more correlated with distant metastases in patients with advanced gynecological cancers, including OC [271].

The majority of cut-off values for NLR are between 2 and 4. However, Forget et al. showed comparable values (0.78 to 3.53) in healthy people [272]. As a result, it is necessary to establish the NLR cut-off values for clinical applications in predicting OC.

In addition to NLR, other neutrophil-related markers have been investigated as potential prognostic biomarkers. Kim et al. found that elevated levels of NET markers (histone–DNA complex, cell-free DNA, and neutrophil elastase) were correlated with worse OS and PFS. Specifically, neutrophil elastase was identified as an independent prognostic factor in patients with HGSOC [273]. Contradictory observations were made by Muqaku et al. indicating that the release of S100A8/A9 protein, associated with the formation of NETs and a high S100A8/CRP ratio, was correlated with a favorable survival of HGSOC patients [274].

**Table 6:** Characteristics of studies on neutrophils and ovarian cancer prognostic significance.

Author/Year	Histology	Stage	Method	Impact	Findings
Kim et al., 2022 [273]	HGSOC	I-IV	ELISA	Detrimental	High circulating levels of NET markers (histone−DNA complex, cell free DNA, and neutrophil elastase) and prekallikrein, correlated with poor OS.
Marchetti et al., 2021 [275]	HGSOC	III-IV	Retrospective study	Detrimental	High NLR correlated with negative PFS and OS.
Henriksen et al., 2020 [245]	EOC	I-IV	FC	Detrimental	High NLR was associated with lack of response to chemotherapy and a poor prognosis.
He et al., 2020 [276]	SOC	I-IV	Retrospective study	Detrimental	Early onset neutropenia correlated with chemosensitivity and favorable PFS and OS.
Singel et al., 2019 [277]	EOC	III-IV	IF	Detrimental	High ascites mtDNA and neutrophil elastase was associated with reduced PFS.
Posabella et al., 2019 [278]	HGSOC	II-IV	IHC	Beneficial	High density of CD66b+ neutrophils was associated with chemosensitivity and longer RFS.
Chen et al., 2018 [279]	n.a	n.a	Meta-analysis	Detrimental	High NLR ratio correlated negatively with PFS and OS.
Komura et al., 2018 [262]	EOC	I–IV	Retrospective study	Detrimental	Pre-treatment neutrophilia and high NLR correlated with poor disease outcome.
Huang et al., 2017 [280]	EOC	I-IV	Meta-analysis	Detrimental	Elevated pre-treatment NLR correlated with poor disease outcome.

EOC various subtypes; HGSOC, high-grade serous ovarian cancer; SOC, serous ovarian cancer; OS, overall survival; PFS, progression-free survival; RFS, recurrence-free survival; IHC, immunohistochemistry; FC, flow cytometry; IF, immunofluorescence; ELISA, enzyme-linked immunosorbent assay; n.a, not available.

### 4.7. Platelets

Platelets have been studied as a potential prognostic marker in OC (Table 7). A high platelet count or thrombocytosis has been wildly associated with a poor disease outcome in patients with OC. Preoperative thrombocytosis is correlated with worse disease-free survival (DFS). Furthermore, in an early disease stage (FIGO I/II), there was a five-fold increase in the risk of death and a nearly eight-fold risk of disease recurrence associated with thrombocytosis [281]. Similarly, in a meta-analysis including a total of 4953 patients with OC pre-treatment, thrombocytosis was significantly associated with OS and PFS [282].

Among 875 patients with HGSOC who underwent primary staging or debulking surgery, thrombocytosis was not found to be a predictor for PFS. However, hyperfibrinogenemia was correlated with OS but not PFS [283]. In a retrospective analysis of 179 women with advanced EOC who underwent cytoreductive surgery and chemotherapy preoperative thrombocytosis, a high platelet ratio and persistent thrombocytosis after chemotherapy combined with high CA-125 levels after chemotherapy were independent poor prognostic factors of OS [284]. In a multicenter case-control study of 1308 patients with advanced and early-stage ovarian clear cell carcinoma (OCCC) and serous ovarian carcinoma (SOC), patients with advanced OCCC were associated with decreased survival outcomes and an increased incidence of venous thromboembolism (VTE) [284].

One of the widely used markers of platelet activity is the platelet-to-lymphocytes ratio (PLR). A high PLR ratio has been associated with a poor prognosis in OC [285]. In patients who underwent debulking surgery, a high PLR had a negative correlation with DFS and OS [286]. Winarno et al. discovered that a high post-operative PLR ratio of patients with an EOC was associated with an increase in the response to platinum-based chemotherapy [287]. Several researchers have also shown that PLR could play a predictive role in the early diagnosis and in distinguishing between benign and malignant tumors [269,270,288,289]. In a cohort of 43 patients, researchers found that preoperative PLR was a predictor for the recurrence of OC [290]. As with NLR, standardized universal cut-off values for PLR are not defined.

**Table 7:** Characteristics of studies on platelets and ovarian cancer prognostic significance.

Author/Year	Histology	Stage	Method	Impact	Findings
Kim et al., 2022 [291]	EOC	III-IV	Retrospective study	Detrimental	Reactive thrombocytosis after cytoreductive surgery and chemotherapy was associated with poor OS and PFS.
Canzler et al., 2020 [292]	EOC	I-IV	Retrospective study	Detrimental	Patients with pretreatment thrombocytosis responded less to treatment and corelated with worse OS and PFS.
Ye et al., 2019 [282]	EOC	I-IV	Meta-analysis	Detrimental	Pre-treatment thrombocytosis corelated with worse OS and PFS.
Yildirim et al., 2015 [269]	n.a	I-IV	Retrospective study	Detrimental	Preoperative high PLR ratio may help identify OC in patients with adnexal masses.
Ma et al., 2014 [293]	EOC	I-IV	Retrospective study	Detrimental	Thrombocytosis and MAR correlated negatively with OS.
Allensworth et al., 2013 [281]	n.a	I-IV	Retrospective study	Detrimental	Preoperative thrombocytosis correlated with worse DFS and OS
Lee et al., 2011 [284]	EOC	III-IV	Retrospective study	Detrimental	Preoperative and post-chemotherapy thrombocytosis correlated with poor prognosis.
Asher et al., 2011 [285]	EOC	I-IV	Retrospective study	Detrimental	Pre-surgery high PLR ratio was associated with poor OS.

EOC various subtypes; OS, overall survival; PFS, progression-free survival; DFS, disease-free survival; MAR, maximal aggregation rate; n.a, not available.

## 5. Conclusions

The interaction between immune cells and platelets in TME is a complex process, and understanding the underlying mechanisms is essential for developing effective therapies. Because of the immunogenic nature of OC, immunotherapy presents a cutting-edge and potential therapeutic approach for the treatment of OC. Even though immunotherapy is evolving quickly and has produced remarkable results, many OC patients continue to experience resistance to it.

Upon arrival, TILs are faced with very immunosuppressive TME due to the presence of an immune resistance mechanism [294,295]. Several mechanisms contribute to evasion, including the presence of immunosuppressive cells (MDSCs, TAMs, or Treg cells), expression of inhibitory molecules (PD-1L, IDO, Arg I, IL-10, B7-H3, TGF-β, PGE2,CTLA-4, and Fas ligand), decreased and/or lost TAA expression, and down-regulation of MHC [296]. Therefore, a deeper understanding of the immune TME of OC can aid researchers in identifying some key sites for innovation that will increase the clinical efficacy of cancer immunotherapy. Therefore, we focused on the mechanisms of OC cells to recruit and re-educate immune cells and how this immunosuppressive network stops immune active cells such as TILs or NK cells from mounting an anti-tumor response.

Both genomic and proteomic methods have been used to discover numerous OC serum biomarkers, which have been assessed alone or in conjunction with CA125 [297,298]. However, the ideal biomarker or combination of biomarkers has not yet been identified. Given that CA125 has been the gold standard for diagnosing OC in the last few decades and considering the advancements in technologies for biomarker discovery, it is necessary to find new markers that may improve CA125’s sensitivity using a combination of hematologic, immunological, and inflammatory markers.

## Figures and Tables

**Table 5:** Characteristics of studies on NK cells and ovarian cancer prognostic significance.

Author/Year	Histology	Stage	Method	Impact	Findings
Webb et al., 2014 [253]	EOC	I-III	IHC, FC	Beneficial	High density of CD103+ NK cells correlated with favorable disease outcome.
Henriksen et al., 2020 [261]	EOC	n.a	FC	Detrimental	A decreased level of NK cell count in recurrent metastasis during chemotherapy is associated with unfavorable prognostic impact.
Henriksen et al., 2020 [245]	HGSOC	I-IV	IHC	Beneficial	High density of CD57+ NK cells correlated with favorable OS.
Krockenberger et al., 2008 [110]	EOC	n.a	IHC, IF	Detrimental	High density of MIF inhibits NK cells and correlated with poor prognosis
Hoogstad-van Evert et al., 2018 [260]	HGSOC	III-IV	FC	Beneficial	High density of CD103+ NK cells correlated with favorable disease outcome.

EOC various subtypes; HGSOC, high-grade serous ovarian cancer; IHC, immunohistochemistry; FC, flow cytometry; IF, immunofluorescence; n.a, not available.

## Data Availability

Data sharing not applicable.
